# Cell Culture Systems for Studying Hepatitis B and Hepatitis D Virus Infections

**DOI:** 10.3390/life13071527

**Published:** 2023-07-08

**Authors:** Grace Sanghee Lee, Michael A. Purdy, Youkyung Choi

**Affiliations:** Division of Viral Hepatitis, National Center for HIV, Viral Hepatitis, STD and TB Prevention, US Centers for Disease Control and Prevention (CDC), Atlanta, GA 30333, USA; qec7@cdc.gov (G.S.L.); mup3@cdc.gov (M.A.P.)

**Keywords:** hepatitis B virus (HBV), hepatitis D virus (HDV), cell culture systems, co-infection and super-infection HBV/HDV

## Abstract

The hepatitis B virus (HBV) and hepatitis D virus (HDV) infections cause liver disease, including hepatitis, cirrhosis, and hepatocellular carcinoma (HCC). HBV infection remains a major global health problem. In 2019, 296 million people were living with chronic hepatitis B and about 5% of them were co-infected with HDV. In vitro cell culture systems are instrumental in the development of therapeutic targets. Cell culture systems contribute to identifying molecular mechanisms for HBV and HDV propagation, finding drug targets for antiviral therapies, and testing antiviral agents. Current HBV therapeutics, such as nucleoside analogs, effectively suppress viral replication but are not curative. Additionally, no effective treatment for HDV infection is currently available. Therefore, there is an urgent need to develop therapies to treat both viral infections. A robust in vitro cell culture system supporting HBV and HDV infections (HBV/HDV) is a critical prerequisite to studying HBV/HDV pathogenesis, the complete life cycle of HBV/HDV infections, and consequently identifying new therapeutics. However, the lack of an efficient cell culture system hampers the development of novel antiviral strategies for HBV/HDV infections. In vitro cell culture models have evolved with significant improvements over several decades. Recently, the development of the HepG2-NTCP sec+ cell line, expressing the sodium taurocholate co-transporting polypeptide receptor (NTCP) and self-assembling co-cultured primary human hepatocytes (SACC-PHHs) has opened new perspectives for a better understanding of HBV and HDV lifecycles and the development of specific antiviral drug targets against HBV/HDV infections. We address various cell culture systems along with different cell lines and how these cell culture systems can be used to provide better tools for HBV and HDV studies.

## 1. Introduction

The hepatitis B virus (HBV) infection causes acute and chronic liver disease and remains a major global health problem [1]. Chronic HBV infection is characterized by the persistence of covalently closed circular HBV DNA (cccDNA) in the nucleus of infected hepatocytes and may lead to fibrosis, cirrhosis [2,3], and hepatocellular carcinoma (HCC) [4]. Current therapeutics for chronic hepatitis B effectively block reverse transcriptase to suppress HBV DNA synthesis but do not eliminate the cccDNA or hepatitis B surface antigen (HBsAg) [5,6]. The molecular mechanisms by which HBV establishes persistent infection remain to be elucidated, but the stability of the HBV genome in the nucleus of hepatocytes is believed to be a key mechanism of HBV’s chronicity [4,7].

The hepatitis D virus (HDV, also known as hepatitis delta), a satellite virus of HBV, is highly pathogenic and requires HBsAg for its propagation and infectivity [8]. There are two major specific patterns of HDV infection. One is the simultaneous infection of a healthy individual with HBV, called a “co-infection”, and the other is “super-infection”, which is the subsequent HDV infection of a patient with chronic hepatitis B [9]. A lack of understanding of virus–host interactions limit the development of efficient curative treatment for HBV/HDV infection. Therefore, a robust cell model could provide useful tools to better understand HBV and HDV life cycles, replication, and interaction with the host. In this review, in vitro, hepatocyte culture systems to support the HBV and HDV lifecycles are discussed to highlight the advantages and the limitations of current systems.

## 2. HBV and HDV Biology

### 2.1. Structure and Genome Organization

**Figure 1:**
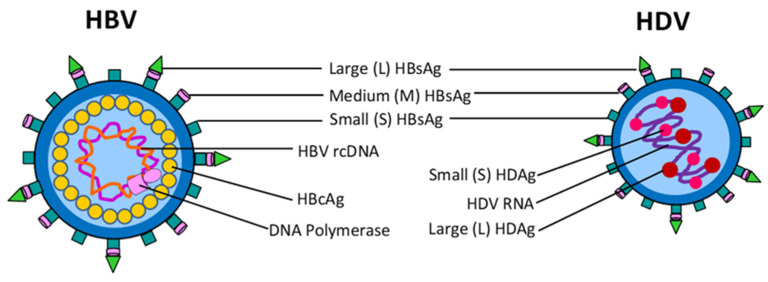
Structure of HBV and HDV virions. Both virions share HBV surface proteins, S-, M-, and L-HBsAg on their envelope. HBV contains a double-stranded DNA genome (or relaxed circular DNA, rcDNA) and the viral DNA polymerase, while HDV has a negative sense single-stranded circular RNA associated with the small delta antigen (S-HDAg) and large delta antigen (L-HDAg).

HBV is a prototype member of the Hepadnaviridae family: an enveloped virus with a 30–42 nm diameter. Ten HBV genotypes (A to J) have been identified [10]. The hepatitis B virion has a spherical, double-shelled structure consisting of lipid-embedded small (S), middle (M), and large (L) hepatitis B surface proteins (HBsAg) that surround an icosahedral inner nucleocapsid composed of the hepatitis B core antigen (HBcAg) [11] (Figure 1). L-HBsAg contains three domains, including pre-S1, pre-S2, and S [12]. M-HBsAg lacks the pre-S1 domain, while S-HBsAg consists of only the S region, which forms a disulfide crosslinked dimer [13]. Pre-S1 and pre-S2 are essential components of the protein envelope of the complete hepatitis B virion [14]. Pre-S1 contains the receptor binding region for HBV [15] and plays important roles in HBV assembly, infection, replication, HBsAg secretion from hepatocytes, and the activation of host immune responses [16,17]. Mutations within the pre-S (pre-S1 and pre-S2) gene can cause immune escape, which affects HBsAg expression and liver disease progression [18]. Point mutations in the start codon of preS2 inhibit the M protein expression completely [19]. In addition, pre-S mutations, including deletions in the pre-S1 and/or pre-S2 region and point mutations, can also be found in patients with chronic hepatitis B (CHB) [20]. Pre-S1 and/or pre-S2 proteins are detected in both HBV-DNA-negative chronic HBsAg carriers in the inactive HbsAg carrier phase and in HBV DNA positive sera in patients in the immune tolerance phase [21], suggesting the presence of pre-S1 and pre-S2 proteins in the serum and liver, which does not reflect the presence of active HBV replication or active liver disease [22,23]. The expression of the HBV core gene produces the core antigen (HBcAg) and the e antigen (HBeAg), which were initiated from different start codons in the same open reading frame (ORF), resulting in two unique homo-dimeric proteins with different structures and functions [24]. HBcAg or HBeAg levels were correlated with HBV DNA [25,26]. Additionally, the nucleocapsid structure of HBcAg is an indicator of viral replication and is the most critical viral factor for HBV clearance in vivo [27]. HBcAg is also associated with HBV-specific T-cell responses [28]. HBcAg is mainly localized in the cytoplasm and is particulate because it is neither secreted nor circulated in the blood. HBeAg is secreted into the blood stream [29,30] and is located between the nucleocapsid core and the lipid envelope. The presence of HBeAg is an indicator of active HBV replication and infection [31,32,33].

HBV has a partially double-stranded and relaxed circular DNA (rcDNA) genome of 3.2 kb whose 5′ ends of the minus strand are covalently attached to the viral polymerase [34]. HBV X protein (HBx) is a multifunctional viral protein that regulates transcriptional activation, cell cycle progression, DNA repair, protein degradation, and several signaling pathways [35]. HBsAg assists with the packaging of the HDV ribonucleoprotein (RNP), leading to HDV assembly and the cellular egress of the virus particle [36]. L-HBsAg, but not M-HBsAg, is essential for HBV and HDV infection [37]. 

HDV is an enveloped small satellite virus with a 35–37 nm diameter and belongs to the genus, Deltaviridae from the family Kolmioviridae [10,38]. It is the smallest known virus to infect humans and requires HBsAg to become virulent [39]. HDV is currently classified into eight species (HDV-1 to HDV-8) [10]. The inner nucleocapsid of the virus contains a negative sense single-stranded circular RNA of 1.7 kb and hepatitis delta antigen (HDAg), which is the only known HDV protein [40] and modulates viral replication through interactions with cellular DNA-dependent RNA polymerases and other host factors [41,42]. There are two forms of HDAg: one is 24 kDa small HDAg (S-HDAg) which participates in virus replication, and the other is 27 kDa large HDAg (L-HDAg) involving virion assembly [43]. HDV is a defective virus that is surrounded by three forms of HBsAg (L, M, and S) and host lipids. Both S- and L-HBsAgs are essential for HDV viral assembly [44], virion packaging, and virus infectivity [37,39,45]. The pre-S1 domain of L-HBsAg is required for HDV cell entry by binding to the sodium taurocholate co-transporting polypeptide (NTCP) receptor, indicating that the viral entry pathways of both HBV and HDV are highly similar. While L-HBsAg is not required for HDV assembly and release, it is essential for HDV infectivity [37]. However, the presence of pre-S proteins and HBsAg in the liver and serum does not indicate the replication of HBV, HDV, and secretion [22,46], suggesting that HDV replication occurs independently from HBV. The HDV genome does not encode its own polymerase but utilizes the host RNA polymerase II the viral replication, while HBV has its own viral polymerase that can be targeted by specific inhibitors [47]. It is wort noting that HDV is the only human viral pathogen that uses the host polymerase [48]. Therefore, HBV therapies based on inhibiting the HBV polymerase cannot directly target HDV infection, which hampers the development of HDV therapeutic targets.

### 2.2. Transmission and Life Cycle

The transmission of both HBV and HDV occurs parenterally through infectious blood or body fluids [49]. While HDV perinatal transmission is rare, HBV can be transmitted from an infected pregnant person to the newborn during pregnancy and childbirth. Thus, perinatal transmission is one of the major sources of HBV transmission [50,51,52]. Since HBV and HDV share the same surface protein (HBsAg) on their virions, the life cycle of HDV is believed to be very similar to that of HBV [53,54,55]. The replication of both HBV and HDV occurs primarily in hepatocytes [40,56,57]. To initiate viral entry and replication, the virions of HBV and HDV bind to the plasma membrane of human hepatocytes through cell-surface factors such as heparan sulfate proteoglycans (HSPGs) [58]. The entry pathway of HBV and HDV into the hepatocytes has not been fully delineated, but HBsAg, especially through the pre-S1 region, plays an essential role in the interaction with the hepatocyte plasma membrane [59,60]. A study identified that the sequence of pre-S1 modulates viral infectivity, where both HBV and HDV infections are dependent on the presence of L-HBsAg [61]. The virus internalizes into host hepatocytes by membrane fusion in an endocytosis-dependent manner [62,63,64]; however, the detailed entry mechanisms of HBV and HDV remain to be determined. HBV and HDV enter hepatocytes through NCTP: a multiple transmembrane transporter at the basolateral membrane of primary hepatocytes. The endocytic transfer of viral particles into the cytoplasm is followed by the release of HBV nucleocapsid and HDV ribonucleoprotein (RNP) complexes from endocytic vesicles, which are subsequently destined to traffic the nucleus [65,66,67]. In the nucleus, the partially double-stranded and relaxed circular DNA (rcDNA) of HBV is converted into cccDNA, which acts as the template for transcription [68,69], while HDV RNP is transported to the nucleus and releases the viral genome, which serves as the template for the transcription of HDV mRNA [40,70,71,72]. 

## 3. Cell Culture Systems for Studying HBV and HDV

Over the last two decades, a series of in vitro cell culture systems have been established to study HBV/HDV lifecycles or host viral immune responses. However, information on the host responses to HBV and HDV infection is limited, and efficient curative treatment for HBV/HDV infections is not available. Restricted host and tissue tropism may be factors behind the lack of robust in vitro cell culture systems for HBV and HDV infection [73]. In vitro cell culture systems have limitations, including modified cell morphology, the lack of an extracellular matrix, deficient accessory cells, the atypical expression of liver enzymes, a lack of proper cell-to-cell communication, short viral infection period, and rapid dedifferentiation (rapid loss of hepatic functions) following isolation and plating [74]. In addition, the innate immune responses against HBV and HDV induce different hepatocyte gene expression profiles [75,76,77,78,79]. The efficient amplification and secretion of progeny viruses are difficult to investigate using cell culture systems [80,81]. The advantages and disadvantages of different cell culture systems in the studies of HBV/HDV infection are discussed below. 

### 3.1. Primary Human Hepatocytes

Primary human hepatocytes (PHHs) are susceptible to HBV/HDV infection [74]. PHHs are used to evaluate hepatic metabolism, drug–drug interactions, and drug toxicity in vitro. They have become the gold standard for a hepatic in vitro culture model [74,82]. PHHs have long been the exclusive hosts of various hepatotropic pathogens, including HBV, HCV, HDV, and Plasmodium parasites [83]. PHHs support the complete life cycle of HBV and HDV infections and exhibit normal hepatic functions such as hepatocyte polarization and bile production [84]. PHHs possess various hepatocyte-specific host factors and exhibit a fully functional innate immune system in response to the infection [76,78,85]. Therefore, PHHs are the most physiologically relevant in vitro culture system for HBV/HDV infection [86]. Notably, NTCP has been identified as the cellular receptor of HBV entry, and HBV particles activated Toll-like receptor signaling upon the infection of PHHs [55,87]. 

The limitations of PHHs for the study of HBV/HDV infection include the limited availability of high-quality donors and life-span [88], variable susceptibility to HBV/HDV infection, the loss of their differentiation functions after plating [74], difficult to manage in culture conditions because of no proliferation in the culture, variable reproducibility between batches [89], and low infection efficiency because of rapid dedifferentiation in the culture [74]. When PHH is co-cultured with nonparenchymal cells, the de-differentiation process is delayed, which prevents its loss of susceptibility to HBV [90,91]. Persistent HBV infections in PHHs are limited to high-quality hepatocyte donor lots, and replication lasts only for 14–19 days post-infection [92]. In microscale conditions, persistent HBV infection is established for 30 days in self-assembling co-cultured primary human hepatocytes (SACC-PHHs) [93]. Additionally, susceptibility to HBV infection in SACC-PHHs is not donor-dependent and can be established without suppressing innate immune signaling where SACC-PHHs persistently support both HBV/HDV coinfection and superinfection for up to 28 days in a micro-scalable system [94]. 

The transcriptomic analysis in traditional PHH culture systems is limited due to rapid de-differentiation and the subsequent loss of infection. However, hepatocytes in SACC-PHHs remain a mature hepatic phenotype regardless of the HBV mono and HBV/HDV co-infection condition [94]. The findings of differential gene expressions indicate no significant differences in genes that are involved in innate immune activation in HBV mono-infection and HBV/HDV co-infection [94], whereas the HDV infection induces the strong activation of IFN-β and IFN-λ in PHHs [95]. PHHs are susceptible to HBV and HDV infection, but limited availability and technical difficulties restrict their use in the HBV/HDV infection model.

### 3.2. HepaRG

The HepaRG cell line, derived from a hepatitis C virus (HCV)-induced liver tumor, is a well-established hepatic cell model [96]. The HepaRG cell line is considered an alternative to PHHs because this cell line expresses transcripts of various nuclear receptors (the aryl hydrocarbon receptor, pregnane X receptor, constitutive androstane receptor, and peroxisome proliferator-activated receptor alpha) and liver-specific enzymes like the major cytochrome P450 (CYP1A2, 2C9, 2D6, 2E1, 3A4) which plays a pivotal role in the detoxification of xenobiotics, cellular metabolism, and homeostasis [97]. Other hepatoma-derived cells, such as Huh7 and HepG2, do not express high levels of CYP450s like HepaRG cells [98,99]. In addition, Phase I and Phase II xenobiotic metabolizing enzymes and membrane transporters are expressed in HepaRG cells, which is comparable to PHHs [99,100]. Thus, HepaRG may have better hepatic functions than other hepatoma cell lines. HBV-infected HepaRG cells produce infectious HBV particles for more than 100 days in a differentiated state [101]. Various CYP450s expressed in differentiated HepaRG cells make them more comparable to cultured PHHs [102,103]. A caveolin-1, one of the plasma membrane components involved in the endocytosis of different ligands and signaling pathways within the cells, is required for productive HBV infection in HepaRG cells [104]. In HepaRG-NTCP cells, HBV evades the induction of interferon (IFN) and the antiviral effects of IFN-stimulated genes (ISG) [105], while HDV efficiently induces IFN-β and IFN-λ responses in differentiated HepaRG cells [79]. HDV replication is recognized by melanoma differentiation-associated protein 5 (MDA5) and induces IFN-β/λ responses but is also insensitive to the IFN-activated stage, where MDA5 depletion has little effect on HDV replication despite dampening the HDV-induced IFN response [95]. Because of these features, HepaRG cells can be used for antiviral drug metabolism and its evaluation [106,107,108].

Despite these extensive research efforts, HBV/HDV infection in HepaRG cells has not been widely studied because HBV infection in HepaRG cells is limited or there is no cell-to-cell spreading. Additionally, HBV infection appears to be a slow process where viral replication starts at around 8 post-infection days and reaches a maximum at day 13 [96,101]. In addition, the conversion of the input relaxed circular viral DNA into cccDNA is inefficient; however, no further amplification of cccDNA has been found to occur in differentiated HepaRG cells [101]. To improve these limitations, dimethyl sulfoxide (DMSO) can be used for the differentiation of HepaRG cells into hepatocyte-like cells where susceptibility to HBV infection is closely correlated to the differentiation status of HepaRG cell lines [96]. As an alternative to DMSO, forskolin promotes functional polarization and differentiation by increasing hepatic markers like cytochrome P-450 [109]. A cocktail of five chemicals (5C-medium), Forskolin (an adenylate cyclase inhibitor) combined with SB431542 (a TGF-β inhibitor), IWP2 (a Wnt inhibitor), DAPT (a Notch inhibitor) and LDN193189 (an inhibitor of bone morphogenetic protein, BMP), accelerate the differentiation of HepaRG cells and have been used for maintaining the differentiated PHH characteristics of HepaRG cells [110]. However, differentiation using the 5C cocktail in HepaRG cells does not increase cccDNA levels or subsequent HBV replication markers, although it improves the efficiency of HDV infection [111].

### 3.3. Huh7

The Huh7 cell is a permanent cell line that is derived from hepatocellular carcinoma and is used as an experimental substitute for primary hepatocytes [112]. However, Huh7 cells only partially mimic normal hepatocytes due to their poor polarization [113]. The pre-S1 domain of the HBV large envelope protein is a key determinant for receptor(s) binding; however, the Huh7 cell line has not been shown to have detectable amounts of the HBV cellular receptor [114]. After identifying hNTCP as a functional receptor for HBV and HDV infection, NTCP-overexpressing Huh7 cells were permissible for HBV and HDV infection and were used for the study of the HBV/HDV lifecycle and specifically for viral entry [115]. Another stable cell line, Huh7-END (Huh7-HDV-Env-NTCP clone B1), also supports the continuous and high-level production of HDV particles and supports the virus spread to co-cultured cells [116]. Since Huh7-END expresses NTCP, it is susceptible to *de novo* HDV entry. Thus, HuH7-END cells could be used for the screening of antiviral drugs targeting HDV replication.

Most in vitro cell culture systems for HBV infection except PHHs require DMSO, but a study has reported that DMSO is not required for HBV infection in Huh7.5-NTCP cells: a derivative of the HuH7 cell line [117]. DMSO supplementation induces cell growth arrest [118], an alteration in protein expression [119], and cytotoxicity [120], resulting in the failure to restore many liver functions. These studies suggest that the elimination of DMSO treatment in the cell culture system could improve HBV infection.

The Huh7 cell line is permissive for HBV replication and viral particle formation but not to events relating to viral uncoating and replication due to a lack of unidentified host factor(s) [121]. In mammalian species, pattern recognition receptors (PRRs) such as the retinoic acid-inducible gene I (RIG-1), MDA5, and Toll-like receptors (TLRs) recognize RNA viruses and play an essential role in the activation of innate immune responses. HDV infection induces the type I interferon response in infected cells; however, these responses are not able to suppress HDV replication or spread [122]. Another study showed that IFN-α-induced intracellular signaling is impaired in HDV-transfected Huh7 cells where HDV subverts the effect of the IFN-alpha by blocking tyrosine kinase 2 (Tyk2) activation and results in the selective impairment of activation and the translocation of the signal transducer and activator of transcription 1 (STAT1) and signal transducer and activator of transcription 2 (STAT2) to the nucleus [123]. The interference of IFN-α signaling by HDV could be an important mechanism of viral persistence and treatment resistance.

### 3.4. HepG2 and HepG2-NTCP

HepG2 cells were isolated from a human hepatocellular carcinoma. HepG2 cell lines are widely used in drug metabolism and hepatotoxicity studies [55,63] even if HepG2 cells exhibit much lower expression levels of many drug-metabolizing enzymes such as cytochrome P450 compared to PHHs [124,125,126]. Two HepG2-derived stably transfected cell lines, HepG2.2.15 [127] and HepAD38 [128], were used to produce cell culture-derived HBV, accessing the late stage of the virus life cycle and antiviral research [129,130,131]. The exogenous expression of NTCP in HepG2 cells supported the entire HBV life cycle and viral spread [121,132]. A commercial human inducible pluripotent stem cell (iPSC)-derived hepatocyte maintenance medium (HMM) was shown to enhance HBV infection and NTCP expression in HepG2-NTCP cells [133]. With features like efficient viral infection and high reproducibility experimental results, HepG2-NTCP cells are a surrogate model for hepatocytes [55,134].

A comparison of gene expression profiles in primary human hepatocytes, including hepatocellular cell lines such as HepG2 and Huh7, and human liver tissue have indicated that hepatocellular cell lines have a highly differentiated nature of hepatocytes compared to liver tissues [125,135,136]. Additionally, NTCP-overexpressed HepG2, Huh7, and undifferentiated HepaRG cells have substantially different infection efficiencies for HBV and HDV. HepG2-NTCP cells appear to be more susceptible to HBV than Huh7-NTCP cells, whereas Huh7-NTCP cells and HepaRG have better infection efficiencies than HDV infection [121]. These studies suggest that additional host factors may be needed for optimal virus infection. HMM activates the cytomegalovirus immediate-early (IE) promoter, which is induced by NTCP expression in the HepG2-NTCP cells and leads to the upregulation of several metabolic pathways. Microarray analysis using HDV- and HBV-infected HepG2-NTCP cells and PHH found that HDV, but not HBV infection, strongly activated a broad range of interferon-stimulated genes (ISGs): IFN-β and IFN-γ; however, HDV replication was mostly insensitive to innate immune responses mediated by MDA5 and was not directly affected by exogenous IFNs after the establishment of the infection [95].

The HepG2-NTCP cell culture system clearly provides useful tools to better understand the life cycles of HBV and HDV infection. However, there are some limitations of HBV and/or HDV infection in HepG2-NTCP cells. Viral replication is limited due to a lack of miRNA-122 in HepG2 because miRNA-122 is the most abundant liver-specific miRNA and plays key roles in liver development and hepatic function. The loss of miRNA-122 was found in liver cancer in HBV-infected livers [137]. The extra use of polyethylene glycol (PEG) and DMSO could restrict the study of HBV/HDV infection pathways by inhibiting the amplification of the viral genome and viral progeny production [138,139,140]. The elimination of the extra usage of PEG and DMSO could improve infection efficiency [141,142,143]. These improvements are desirable to more closely mimic physiological conditions.

### 3.5. NCTP-Expressed Hepatoma Derived HepG2 Cell Lines

Recently developed HepG2-NTCP sec+ cells support the complete HBV life cycle, long-term viral spread, and amplification of HBV derived from patients’ serum samples; however, it does not induce significant gene expression changes in HepG2-NTCP sec+ [144]. However, HepG2-NTCP sec+ cells have a short-distance route for HBV spread to neighboring cells, causing HBV-infected cell clusters and requiring high viral titer inoculum (up to 5000 GEq/mL) as well as PEG to increase infectivity. Considering the practical disadvantages of HepG2-NTCP cells, the HepG2-NTCP-A3/C2 subclone has relatively increased viral production under PEG-free conditions and showed an improved in vitro HBV infection system [145]. Additionally, hepatocyte nuclear factor 4α and the NTCP-expressing hepatoma cell line, BEL7404, have been permissive for HBV replication and susceptible to HBV infection [146]. Nevertheless, more cell culture models with increased susceptibility to HBV infection need to be identified and studied.

### 3.6. The 3D Culture

The in vitro culturing and maintenance of hepatocytes are difficult because they are rapidly losing their cuboidal morphology and liver-specific functions [147,148]. The elongated spindle morphology of hepatocytes has been observed in 2D-cultured PHH monolayers [149,150]. To maintain an intact morphology, 3D-cultured PHHs have developed a spheroid morphology [151]. The most common 3D culture method is placing PHHs on a single-layer collagen matrix; however, basic liver functions decrease in this cell culture system after a week [152]. To prolong and enhance hepatic functions in vitro, a 3D cell culture was developed by fabricating PHH microtissues using droplet microfluidics encapsulated with fibroblasts [153]. This new system displayed the stable expression of hepatocyte genes and maintained functional liver-specific genes for a month or longer. In addition, lower levels of apoptotic markers were detected in 3D-cultured PHHs compared to 2D-cultured PHHs [154,155]. The 3D cultures of Huh7 cells exhibited improved hepatocyte differentiation [156,157]. The 3D-cultured PHHs do not need either PEG or DMSO for HBV infection [86], although no differences were seen in cytochrome P450 enzyme levels between 2D- and 3D-cultured PHHs [155]. However, maintaining a natural hepatic environment and liver-specific functions such as the expression of albumen, transferrin, apoptotic-related markers, and metabolizing enzymes in cell culture systems is still greatly desired.

## 4. Conclusions and Future Developments

A robust in vitro cell culture system supporting HBV and/or HDV infection is essential to study life in the cycles of HBV and/or HDV and to further develop new therapeutic approaches. The development of cell culture systems using PHH, HepaRG, Huh7, and HepG2 cells has significantly contributed to studying the molecular mechanisms that underlie HBV and HDV infections. The discovery of NTCP as an entry receptor of HBV and HDV has led to the establishment of in vitro cell culture systems for HBV and HDV infection [55,121]. Despite these advances, HBV and HDV research is still limited because of the lack of experimental models supporting native HBV and/or HDV infections (Table 1). Culturing hepatocytes supporting HBV/HDV replication and maturation has limitations such as a lack of complete HBV/HDV life cycles, low infection efficiency, no viral spread, the use of polyethylene glycol (PEG) during entry to improve glycosaminoglycan-dependent binding [142], the use of DMSO to enhance HBV infection [143], and low cccDNA production rates [158,159]. The ultimate goal of in vitro cell culture study is to establish standardized and reproducible conditions for HBV/HDV infection, the better characterization of viral progeny, and increased cccDNA production, which is needed for the development of a cure for chronic hepatitis B infection.

## Figures and Tables

**Table 1:** Advantages and limitations of the cell culture system supporting HBV and/or HDV infection.

Cells	Advantages	Limitations
PHH	• Supports the complete life cycle of HBV and HDV infections	• Limited availability of high-quality donors and lifespan
	• Contains various hepatocyte-specific host factors	• Variable susceptibility to HBV/HDV infection
	• Exhibits a fully functional innate immune system	• Loss of their differentiation functions after plating
		• Difficult to manage in cultural conditions
HepaRG	• Contains hepatic functions	• Low infection efficiency
	• Expresses transcripts of various nuclear receptors	• Requires differentiation
		• Limited cell-to-cell spreading
Huh7-NTCP	• Better infection efficiencies for HDV infection	• Partially mimic normal hepatocytes due to poor polarization
		• No detectable amounts of the receptor
HepG2-NTCP	• Readily available	• Partially mimic normal hepatocytes
	• High reproducibility	• Low infection efficiency/limited viral replication
	• Robust viral infection	• Require extra usage of PEG and DMSO
HepG2-NTCP sec+	• Supports the complete HBV life cycle	• Require high viral titer inoculum
	• Long-term viral spread	• Require PEG to increase viral infectivity
The 3D culture	• Maintains an intact morphology	• Does not fully maintain a natural hepatic environment and liver-specific functions
	• No PEG or DMSO needed	

## Data Availability

All data generated or analyzed during this study are included in this article.
